# Biotic Control of Surface pH and Evidence of Light-Induced H^+^ Pumping and Ca^2+^-H^+^ Exchange in a Tropical Crustose Coralline Alga

**DOI:** 10.1371/journal.pone.0159057

**Published:** 2016-07-26

**Authors:** Laurie C. Hofmann, Marguerite Koch, Dirk de Beer

**Affiliations:** 1 Microsensor Group, Max Planck Institute for Marine Microbiology, Bremen, Germany; 2 Aquatic Plant Ecology Lab, Biological Sciences Department, Florida Atlantic University, Boca Raton, Florida, United States of America; University of California, Merced, UNITED STATES

## Abstract

Presently, an incomplete mechanistic understanding of tropical reef macroalgae photosynthesis and calcification restricts predictions of how these important autotrophs will respond to global change. Therefore, we investigated the mechanistic link between inorganic carbon uptake pathways, photosynthesis and calcification in a tropical crustose coralline alga (CCA) using microsensors. We measured pH, oxygen (O_2_), and calcium (Ca^2+^) dynamics and fluxes at the thallus surface under ambient (8.1) and low (7.8) seawater pH (pH_SW_) and across a range of irradiances. Acetazolamide (AZ) was used to inhibit extracellular carbonic anhydrase (CA_ext_), which mediates hydrolysis of HCO_3_^-^, and 4,4′ diisothiocyanatostilbene-2,2′-disulphonate (DIDS) that blocks direct HCO_3_^-^ uptake by anion exchange transport. Both inhibited photosynthesis, suggesting both diffusive uptake of CO_2_ via HCO_3_^-^ hydrolysis to CO_2_ and direct HCO_3_^-^ ion transport are important in this CCA. Surface pH was raised approximately 0.3 units at saturating irradiance, but less when CA_ext_ was inhibited. Surface pH was lower at pH_SW_ 7.8 than pH_SW_ 8.1 in the dark, but not in the light. The Ca^2+^ fluxes were large, complex and temporally variable, but revealed net Ca^2+^ uptake under all conditions. The temporal variability in Ca^2+^ dynamics was potentially related to localized dissolution during epithallial cell sloughing, a strategy of CCA to remove epiphytes. Simultaneous Ca^2+^ and pH dynamics suggest the presence of Ca^2+^/H^+^ exchange. Rapid light-induced H^+^ surface dynamics that continued after inhibition of photosynthesis revealed the presence of a light-mediated, but photosynthesis-independent, proton pump. Thus, the study indicates metabolic control of surface pH can occur in CCA through photosynthesis and light-inducible H^+^ pumps. Our results suggest that complex light-induced ion pumps play an important role in biological processes related to inorganic carbon uptake and calcification in CCA.

## Introduction

Crustose coralline algae (CCA) are a cosmopolitan group of red calcifying macroalgae (Rhodophyta) that provide critical habitats for a diversity of marine organisms [1–4] and facilitate settlement for many benthic larvae [5–7], including new coral recruits on reefs [8,9]. Most recently, the application of long-lived coralline algae in paleo reconstructions of past climates [10–12] broadens the significance of their natural longevity attributed to their indeterminate growth pattern. Due to the ecological and physical importance of CCA to marine ecosystems, and their paleontological significance for climate reconstructions, the potential loss of this diverse and ubiquitous group to global change (increasing temperature and pCO_2_) is a major concern [13–18]. For calcifying organisms, such as CCA, global change is likely to have significant impacts on their ability to calcify, but conflicting results make it difficult to predict if CCA, as a group, will be able to accommodate increasing surface ocean temperature and pCO_2_ (e.g. [13,19–24]). Therefore, a more comprehensive understanding of the mechanisms of CCA calcification is needed.

Primary calcification in some CCA occurs in surface epithallial cells and in a meristematic cell layer between the upper epithallial cells or filaments and the lower perithallial cells or filaments [12], indicating that for some species calcification is occurring in cells several layers removed from the influence of external bulk seawater. There is also evidence that calcite crystal formation in the cell wall depends principally on cell membrane ion pumps [25] and nucleation by organic material within the cell wall [21–22, 25–26]. Despite the likely role of active ion pumps in crystal formation, calcification has been shown to occur in darkness [12] and in non-photosynthetic branching tips [19], and thus may not be directly coupled to photosynthesis, or may be dependent on photosynthate translocation [26]. Pueschel et al. [25] present data supporting metabolic control of calcification and decalcification of epithallial cells necessary for new cellular growth beyond older calcified cells in the epithallial region [27]. They clearly show using transmission electron microscopy (TEM), that organic microfibrils of the cell wall produce ingrowths that they suggest increase the surface area for [H^+^] flux and controls decalcification. Adey et al. [12] also contend that CCA calcification and decalcification are highly controlled metabolic processes incorporating complex ion transport systems. These processes support CaCO_3_ growth associated with the cell wall and formation of reproductive structures that necessitate the dissolution of several adjacent calcified vegetative cells. Further, once reproduction occurs, cell walls of the empty reproductive structure calcify, and centers infill with calcified material, some with diverse carbonate minerals, including dolomite, potentially more resistant to high pCO_2_ than high magnesium calcite [21–22]. While Adey et al. [12] advocate that cell wall calcification is internally controlled, there is secondary calcification between cell walls within the perithallial region that may be associated with photosynthesis, evidenced by the fact that there is a larger area of disorganized secondary crystals under greater irradiance and temperature. The pattern of CCA calcification appears analogous to the highly oriented aragonite crystals associated with the cell wall of the Chlorophyte macroalgal genus, *Halimeda*, in contrast to the adjacent randomly oriented crystals in spaces between filaments [28–30]. Different modes of calcification driven by diverse processes complicate identifying mechanisms of CCA calcification and may account for some discrepancies (i.e. strong negative effect, no effect, acclimation) on the pCO_2_ effects of CCA calcification in the ecological literature (e.g. [13,19–24]).

The presence of diffusive boundary layers (DBL) also plays a role in calcification and photosynthesis at the algal thallus surface. Recent research has shown that macroalgae influence the thallus surface pH through their metabolic activity [31–33]. The metabolically-induced changes in surface pH are partially a result of photosynthesis and respiration, which affect carbonate chemistry [28,34–37]. The consumption and release of CO_2_ in the light and dark results in an increase and decrease, respectively, in surface pH. This change in pH can result in surface chemistry that is profoundly different from that in the water column, depending on flow conditions. Thus the DBL may ameliorate potential effects of high pCO_2_, particularly under low flow regimes where a thick DBL develops [32–33]. The photosynthesis-induced increase in pH is thought to favor calcification due to the associated increase in aragonite saturation state and increase in CO_3_^2-^ ions [30,33]. This positive relationship between photosynthesis and calcification has been observed in a diversity of calcareous macroalgae at ambient seawater pH, including CCA [38–41].

A recent model of CCA light calcification suggests that the biological control over calcification under high irradiance may prevent dramatic CCA responses to the expected decline in surface ocean pH [42]. The model assumes that HCO_3_^-^/H^+^ symporters transport these ions into the cell, where they are catalyzed to CO_2_ and H_2_O by internal carbonic anhydrase (CA), thus providing much of the CO_2_ needed for photosynthesis. During photosynthesis and CO_2_ fixation, OH^-^ is released, which increases pH at the surface of the cells and consequently facilitates precipitation of CaCO_3_. In the dark, CA catalyzes the hydrolysis of respiratory CO_2_ to HCO_3_^-^ and H^+^, which are buffered by CO_3_^2-^. Presently, the specific role of irradiance in this model, and if it primarily drives photosynthetic processes that are directly coupled to calcification, or active transport systems of other ions (i.e. proton pumps) requiring ATP, is not clear. Recent advances in the most well-studied algal-carbonate system, the phytoplankton cocolithophores [43], and previous studies on macroalgae (reviewed in [15,16,44]) suggest that calcification in marine photoautotrophs are likely complex involving multiple processes and a diversity of ion transport systems.

In order to better understand the relationship between inorganic carbon uptake pathways, photosynthesis and calcification at a micro-scale, we designed microsensor experiments to measure pH, O_2_ and Ca^2+^ directly at the surface of a tropical CCA from the Florida Keys. We investigated the effect of a range of irradiances on O_2_, and Ca^2+^ fluxes and surface pH at two seawater pH levels. Further, photosynthesis and two metabolic pathways of inorganic carbon uptake were inhibited in order to investigate how these processes affect surface pH and O_2_ and Ca^2+^ fluxes at varying irradiance and seawater pH levels. We expected that Ca^2+^ uptake would be greatest under high irradiance, coincident with elevated pH at the thallus surface, and this relationship would be disrupted upon inhibition of photosynthesis. Further, we hypothesized that inhibition of inorganic carbon uptake would decrease surface pH, O_2_ and Ca^2+^ fluxes. Finally, we hypothesized that at low pH, Ca^2+^ would efflux upon dissolution in the dark.

## Materials and Methods

### CCA Collection, Acclimation and SEM Imagery

The CCA examined were collected (March 9, 2015) from the Lower Florida Keys in a hard-bottom patch reef (ca. 4 m) south of Big Pine Key (24°37.371’ N, 81°21.706’ W; permit FKNMS-2015-032). Specimens were transported to the Max Planck Institute for Marine Microbiology in Bremen, Germany, where they were acclimated in artificial seawater (Tropic Marine Pro-Reef Salt in de-ionized water to 35 psu; see [45] for specific chemistry) at 27°C under 200 μmol photons m^-2^ s^-1^ on a 12 hour day/night cycle. This light intensity was saturating for photosynthesis, despite higher light intensities measured at the collection depth in the field (700–1200 μmol photons m^-2^ s^-1^). The light source consisted of an HQI lamp fixed above the aquarium. The seawater temperature was maintained in a temperature-controlled room, and a cooling thermostat (Julabo, Seelbach, Germany) pumped cooled water through a cooling spiral in the header tank of the recirculating system to offset heat produced by the lamp. Four separate specimens subsampled from the same rock were used for experiments, and are referred to throughout the manuscript as CCA I-IV.

SEM imagery was conducted to identify the CCA specimens. Prior to SEM imagery, tissue sub-samples of the CCA examined were dehydrated through graded ethanol concentrations (20–100%) and stub-mounted after cross sectioning to exposed interior at the surface. The mounted sections were sputter coated with a palladium target and observed on an FEI XL 30 Field Emission ESEM/SEM (Advanced Microscopy Lab University of Miami)

**Fig 1:**
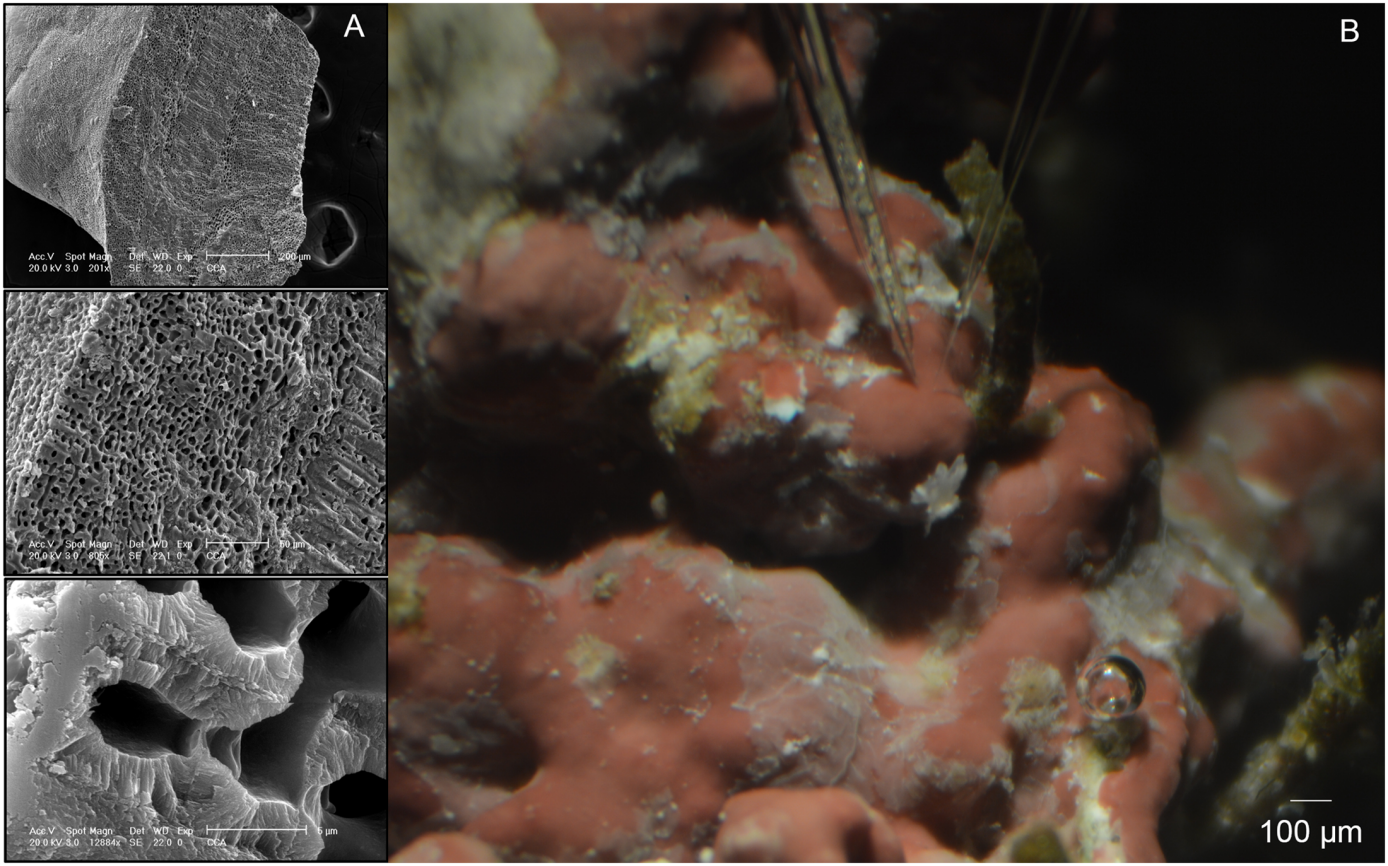
Identification of CCA and microsensor set-up. A) Scanning electron micrographs of the tropical CCA examined in physiological experiments at 201, 805 and 12,884X (from top to bottom) showing calcite structure of the thalli and B) two microsensors at the thallus surface (note white patches where surface cells are sloughing and neighboring epiphyte-free surfaces).

Unfortunately, identification of the CCA to species was not possible because reproductive structures were not present [46]. However, we acquired scanning electron micrographs (SEM) of the individual rock (Fig 1a, S1 Fig) and describe its characteristics, which are most similar to *Porolithon* sp. The CCA had a thick (~600 **μ**m) multi-cellular thalli with highly calcified cell walls throughout. Further, there were no observable trichocytes in the SEM images taken in cross section. Internal cells were vertically aligned, but rows poorly defined. Below the first cell layers, fusions were noted between cells. It was also apparent that surficial cells were being sloughed, as noted throughout the study. This turnover of cells is clearly depicted in Fig 1b. Because of the uncertainty in identifying CCA without reproductive structures, we describe our study organism throughout this manuscript as a tropical CCA, rather than ascribing it to a specific genus or species.

### Experimental Set-up

For microsensor measurements, CCA specimens of approximately 10 cm^2^ were placed into a plexiglass flow cell (19 cm x 9 cm x 9.5 cm) attached to a reservoir. Water was pumped longitudinally through the chamber at a rate of approximately 2 cm s^-1^ (judged by particle movement) and passed through a perforated wall (diffusor) at the entrance and exit of the flow-chamber in order to create laminar flow at the thalli surface. The outflow water was recycled via a 1.5 L aerated reservoir. The outflow rate was maintained at approximately 0.004 L s^-1^, resulting in a four-minute residence time in the flow chamber. The total volume of the flow chamber and the reservoir was 2.5 L. Seawater temperature in the flow chamber was maintained at 27°C with a Julabo warming thermostat (Julabo, Seelbach, Germany). The light source used during incubations was a fiber optic halogen lamp (Schott KL1500, Schott, Mainz, Germany) and the down-welling photosynthetically active radiation (PAR) at each light level (1–5) was measured using a LI-190 Quantum Sensor attached to an LI-COR light meter (LI-COR Biosciences, Lincoln, Nebraska, USA). The PAR intensities used during the incubation experiments consisted of five levels between 0–1400 (0, 20, 100, 280, 800, 1400) μmol photons m^-2^ s^-1^.

### Microsensor Construction

Liquid ion exchange (LIX) calcium and pH microsensors with tip diameters of 10–20 μm and fast-responding O_2_ microsensors with a tip diameter of 10 μm were prepared, calibrated and used as described by [47–49]. The LIX sensors were calibrated using pH_NST_ 7.0 and 9.0 buffers (Fluka Analytical, Buchs, Switzerland), O_2_ microsensors were calibrated using air-saturated seawater and anoxic seawater produced by flushing with nitrogen gas. The LIX sensors were connected to a reference electrode (REF401, Radiometer Analytical, Hach Company) and millivoltmeter, while the O_2_ microsensors were connected directly to a picoammeter. Data acquisition and microsensor positioning was performed by a custom built program (μ-profile, Dr. L. Polerecky) using a laptop computer.

### Microsensor Measurements

During all incubations, two microsensors (pH and O_2_ or Ca^2+^ and O_2_) were used simultaneously and were positioned at the surface of the CCA in the flow chamber using motorized micromanipulators (Pyro-Science GmbH, Aachen, Germany). The position of each sensor tip was determined using an adjustable stereomicroscope. Once the tip of the sensor reached the surface of the CCA thallus, this position was set to zero.

#### O_2_, pH and Ca^2+^ surface dynamics

For dynamics studies, simultaneous measurements of paired microsensors (O_2_, pH, and Ca^2+^) were taken with electrodes at the thallus surface. Measurements were taken upon illumination (20, 100 or 800 μmol photons m^-2^ s^-1^) and darkening by recording the sensor signal every second until the system reached steady state. For dynamics studies, the zero position at the surface was kept constant and the sensors were not moved.

#### Microprofiles and interfacial fluxes

Microprofiles of O_2_, Ca^2+^ and pH were obtained by increasing the microsensor distance away from the thallus to 0.8 mm in 50 μm steps. Profiles were replicated four times at each light intensity and these data are presented as averages with standard errors. Interfacial fluxes (J) of O_2_ and Ca^2+^ were calculated from the concentration profiles using Fick’s first law:

$$J=D\;\left(\frac{dc}{dx}\right)$$ (1)

where D is the diffusion coefficient and dc/dx is the concentration gradient in the mass boundary layer above the CCA surface calculated by the change in concentration (c) divided by the change in distance (x). The diffusion coefficient of O_2_ at 27° and 35 psu (2.35 x 10^−9^ m^-2^ s^-1^) was used for net photosynthesis (O_2_ flux) calculations.

#### Gross photosynthesis calculations

Gross photosynthetic rates (GP) were estimated using the rapid light-dark shift method described by [50–51]. The light-dark shifts were repeated five times at each light intensity (0, 20, 100, 280, 800, 1400 μmol photons m^-2^ s^-1^) to obtain an average rate. The diffusion coefficient of calcium with HCO_3_^-^ as a counter ion was calculated to be 1.01x10^-9^ m^-2^ s^-1^ using the self-diffusion coefficients of calcium and HCO_3_^-^ (the most abundant DIC species) at 25°C from [52]. Photosynthesis (P)–irradiance (I) curves were plotted for net and gross photosynthesis and fitted to the equation:

P=Pmax (1−exp(−bI)) (2)

using non-linear regression analysis to estimate the unknown parameters P_max_ and *b* (Sigma Plot 12.0). The Photosynthetic parameters (maximum net photosynthetic rate, P_max_, photosynthetic efficiency, alpha, and light compensation points, I_c_) were determined from Eq 2. Alpha was calculated as *b* x P_max_.

We calculated the minimal distance from the algal surface at which photosynthesis was occurring using the equation:

$$x^{2}\;=qDt$$ (3)

where x = distance (m), D = the diffusion coefficient of O_2_ at experimental temperature and salinity (see above), q = 1 due to the flat surface of the alga, and t = the time (s) required to measure a decrease in O_2_ concentration after turning off the light during the rapid light-dark shifts. Although a decrease in O_2_ was observed immediately, the highest resolution of time measurements we could achieve was 1s.

#### Inhibition of inorganic carbon uptake pathways

Three metabolic inhibitors were used to examine the effects of inhibiting two pathways of inorganic carbon uptake and direct inhibition of photosynthesis. Acetazolamide (AZ), a membrane impermeable carbonic anhydrase inhibitor, was used to investigate the effect of external carbonic anhydrase (CA_ext_) inhibition on surface pH, O_2_, and Ca^2+^ at ambient (8.12) and low (7.80) pH. AZ was dissolved in a 10 mM NaOH solution to a concentration of 0.05 M and, after dissolution, titrated with HCl to a pH of 8.5. The algae were exposed to a final concentration of 200 μM acetazolamide (AZ).

The seawater pH was lowered by adding aliquots of seawater that had been previously acidified with pure CO_2_. Slight changes in seawater pH caused by AZ addition were neutralized with HCl before the CO_2_ saturated seawater was added. The seawater pH was monitored during the incubations with a pH electrode connected to a portable pH meter (WTW GmbH, Weilheim, Germany).

All measurements were made under steady state conditions, which occurred within minutes after changing the light intensity or seawater pH. Microprofiles across a range of irradiances and dynamics upon illumination and darkening were measured with pH, O_2_ and calcium microsensors as described above. Data from rapid light-dark shifts at multiple irradiances were used to determine gross photosynthesis calculated as described above. The microprofiles, dynamics and rapid light-dark shifts were measured first under control conditions (pH 8.12), then under low pH (pH 7.80), then under control conditions + AZ and finally under low pH conditions + AZ. Oxygen and pH or Ca^2+^ and pH were always measured simultaneously. Incubations with AZ at two pH levels were repeated many times, but due to the fragility and sensitivity of the sensors, a complete data set from start to finish was only obtained in two independent experiments with two separate individuals (CCA I & CCA II).

A cellular anion permeability inhibitor, 4,4'- diisothiocyanatostilbene-2,2'-sulfonic acid (DIDS), was used to determine if active HCO_3_^-^ transport occured in this CCA. The DIDS was dissolved in distilled water by heating. A final concentration of 125 μM was added to the circulating incubation system, and gross photosynthesis at all light levels was measured before and after addition of DIDS. A complete data set for this experiment was obtained from a single individual (CCA III).

The herbicide 3-(3,4-Dichlorophenyl)-1,1-dimethylurea (DCMU), which shuts down photosystem II of the light harvesting complex, was also used to confirm the ability of CCA to pump protons across the cell membrane in the absence of photosynthesis. DCMU (0.05 M in ethanol) was added several times in 40 μl aliquots (starting DCMU concentration 1 μM, ethanol 0.017%) to the flow chamber (total reservoir volume decreased to 2 L) while the O_2_ and pH dynamics at the algal surface were recorded before and after each addition in the light and in the dark. Profiles were also performed in order to estimate interfacial fluxes of O_2_. A complete data set for this experiment was obtained from a single individual (CCA IV).

## Results

### O_2_, pH and Ca^2+^ Surface Dynamics

**Fig 2:**
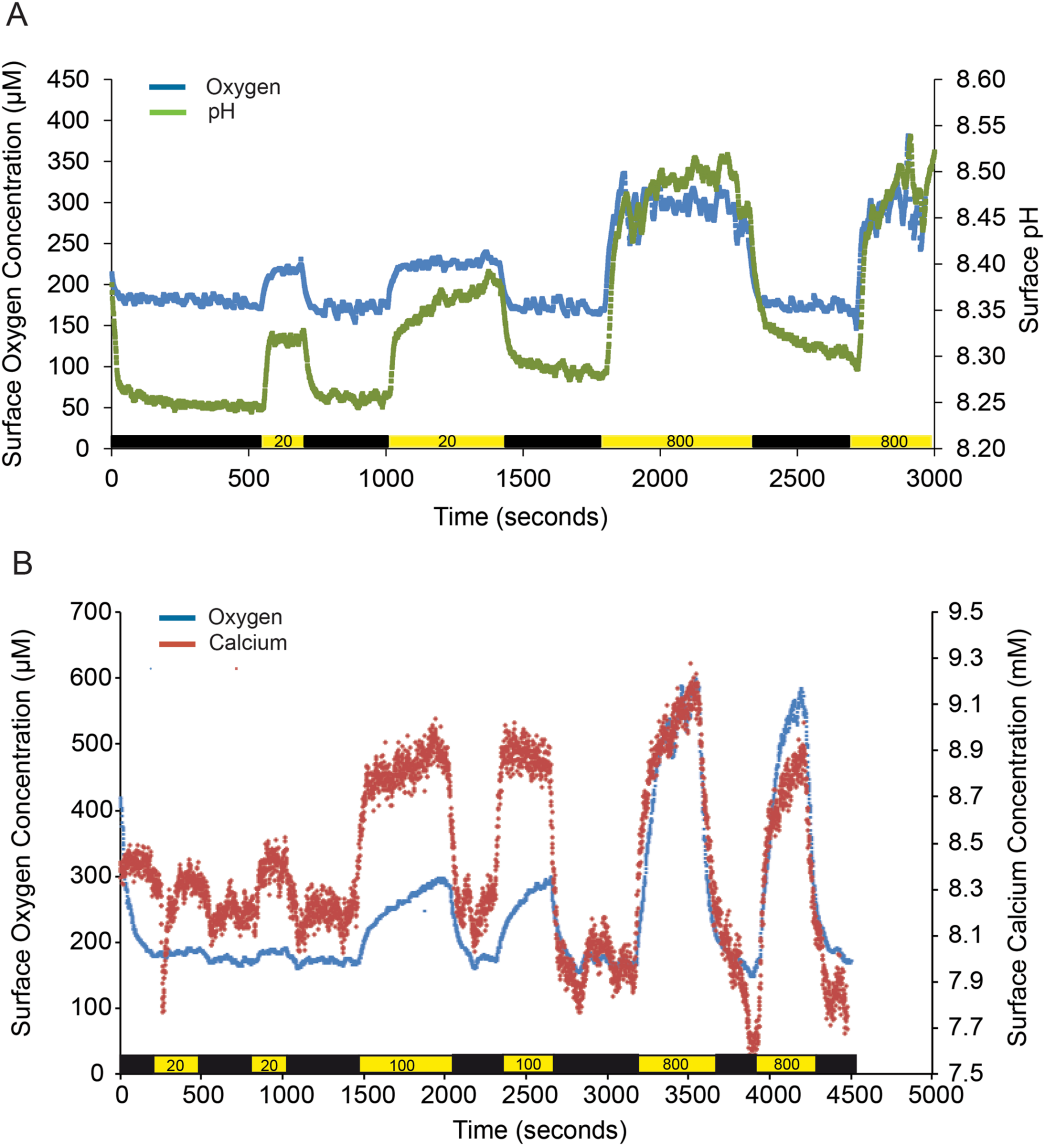
O_2_, pH and Ca^2+^ dynamics. Simultaneous A) oxygen (blue) and pH (green) dynamics and B) oxygen (blue) and calcium (red) dynamics measured at the surface of a single individual (CCA III) at 20 and 100 and 20, 100 and 800 μmol photons m^-2^ s^-1^ light intensities, respectively. The light intensities are indicated in the yellow boxes along the x-axis, and the black boxes indicate darkness. Note the lengths of the x-axes differ.

Simultaneous measurement of pH and O_2_ dynamics at the thallus surface upon illumination and darkening at approximately 10 min intervals showed pH and O_2_ elevation in the light, and reduction in the dark (Fig 2a). Using Eq 3, we calculated that oxygen was produced within the first three to six cell layers (maximum depth of 50 μm below the surface), based on cell size from our SEM images. The rate of both the pH and O_2_ increase or decrease upon illumination or darkening, respectively, was extremely fast, reaching steady state in < 1 min. This rapid response indicated that the pH dynamics must have been partially biologically controlled, rather than an indirect consequence of carbonate chemistry change due to CO_2_ uptake during photosynthesis. The slope of the change in O_2_ and pH dynamics was steeper after the light was turned off compared to after the light was turned on, so that steady state was reached slightly faster in the dark than in the light.

**Fig 3:**
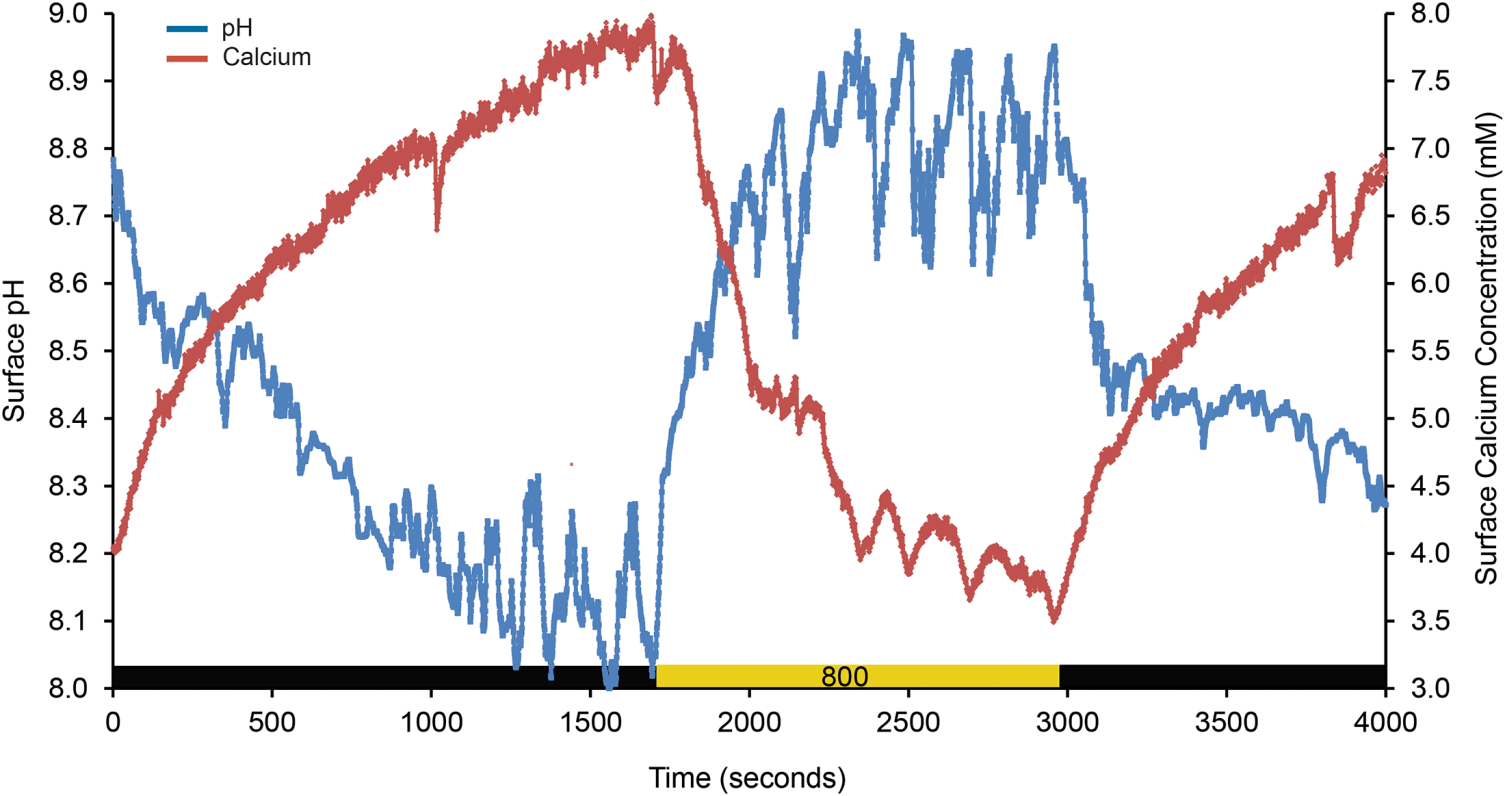
Variable Ca^2+^ dynamics. A dynamic study where a decrease in surface pH (blue) was observed simultaneously with an increase in surface calcium concentration (red) in the dark, and an increase in surface pH and decrease in surface calcium concentration in the light (800 μmol photons m^-2^ s^-1^). Data are from a single individual (CCAII).

Simultaneous measurements of Ca^2+^ and O_2_ dynamics at the algal surface showed a similar pattern (Fig 2b), and simultaneous measurement of Ca^2+^ and pH (data not shown) dynamics also showed that both parameters increased in the light and decreased in the dark. Consequently, there was a net flux of Ca^2+^ out of the cell (Ca^2+^ increase at the surface) and protons into the cell (pH increase at the surface) in the light, and a net flux of Ca^2+^ into the cells and H^+^ out of the cells in the dark. However, on a few occasions, we measured the opposite trend for Ca^2+^, such that the Ca^2+^ concentration at the surface decreased in the light relative to in the dark (Fig 3). In general, the magnitude of change in Ca^2+^ concentration upon illumination and darkening was very large (> 1 mM). The observation that Ca^2+^ fluxes can switch directions in the light and dark suggest the presence of both light-induced Ca^2+^/H^+^ exchange and a H^+^-independent Ca^2+^ channel, or other mechanism controlling Ca^2+^ flux, in the CCA examined.

### Microprofiles and Interfacial Fluxes

**Fig 4:**
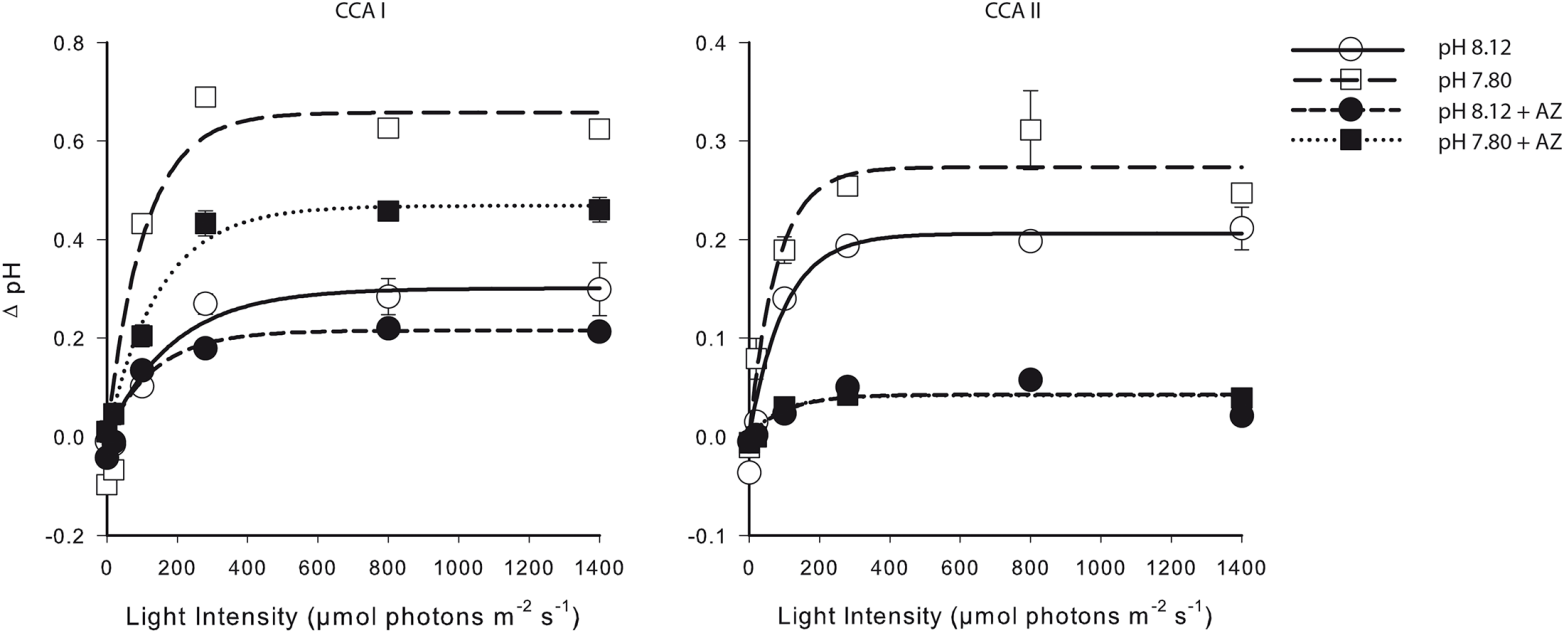
pH gradient is greater at low pH, and inhibited by AZ. The pH gradient (ΔpH = surface pH−seawater pH) as a function of light intensity of two specimens (left: CCA I, right: CCA II) exposed to two seawater pH levels (circles: 8.12, squares: 7.80) in the presence (filled symbols) and absence (open symbols) of the external carbonic anhydrase inhibitor, acetazolamide. Note the scales on the y-axis differ. Data are the mean ± SE of multiple measurements made on the same individuals. Non-linear regression analysis was done using Sigma Plot 12.0 and the equation y = a*(1-exp(-b*x)).

**Fig 5:**
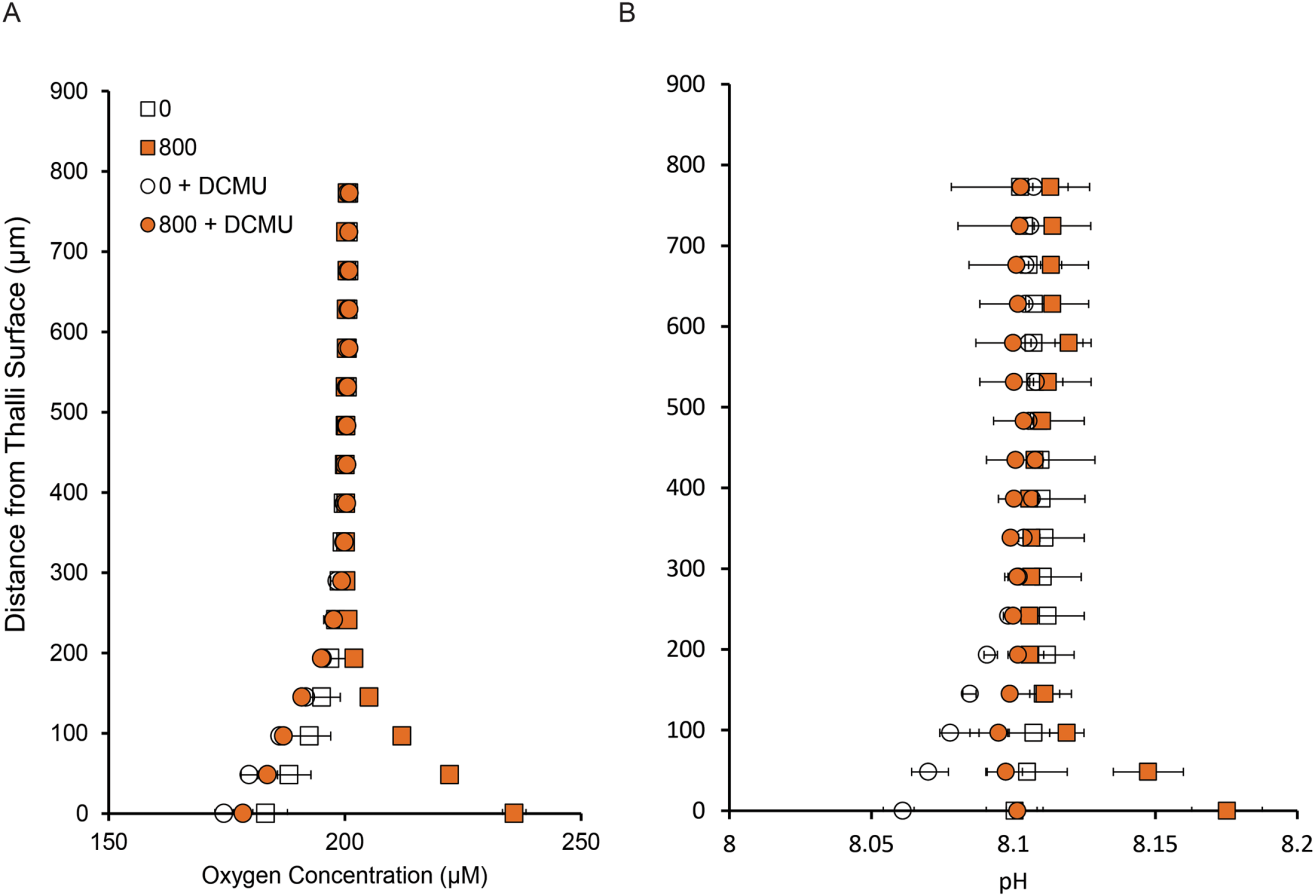
Incomplete inhibition of surface pH elevation by DCMU suggests presence of light-induced proton pump. Profiles of a) oxygen and b) pH from 0–800 μm from the thallus surface in the light (800 μmol photons m^-2^ s^-1^; orange symbols) and dark (white symbols) under control conditions (squares) and in the presence of the herbicide 3-(3,4-Dichlorophenyl)-1,1-dimethylurea (DCMU; circles). Data are means ± SD of multiple profiles measured on a single individual (CCA IV).

Microprofiles of pH under a range of irradiance levels showed that the pH gradient (ΔpH) at the thallus surface was higher at low seawater pH (7.80) than at ambient seawater pH (8.12) at all light intensities investigated in the absence of AZ (Fig 4). This resulted in a similar surface pH regardless of seawater pH treatments. The presence of AZ decreased the ΔpH relative to the respective controls in both pH_SW_ treatments. Inhibition of photosystem-II by the addition of DCMU reduced net photosynthesis at saturating light (800 μmol photons m^-2^ s^-1^) by 141 ± 6.4%, and increased dark respiration by 56 ± 18% (Fig 5a). The pH gradient also decreased in the dark, but was not completely inhibited in the light (inhibition of ΔpH by DCMU was 90%, Fig 5b), suggesting the presence of a photosynthesis-independent, light-induced proton pump driving proton uptake.

**Fig 6:**
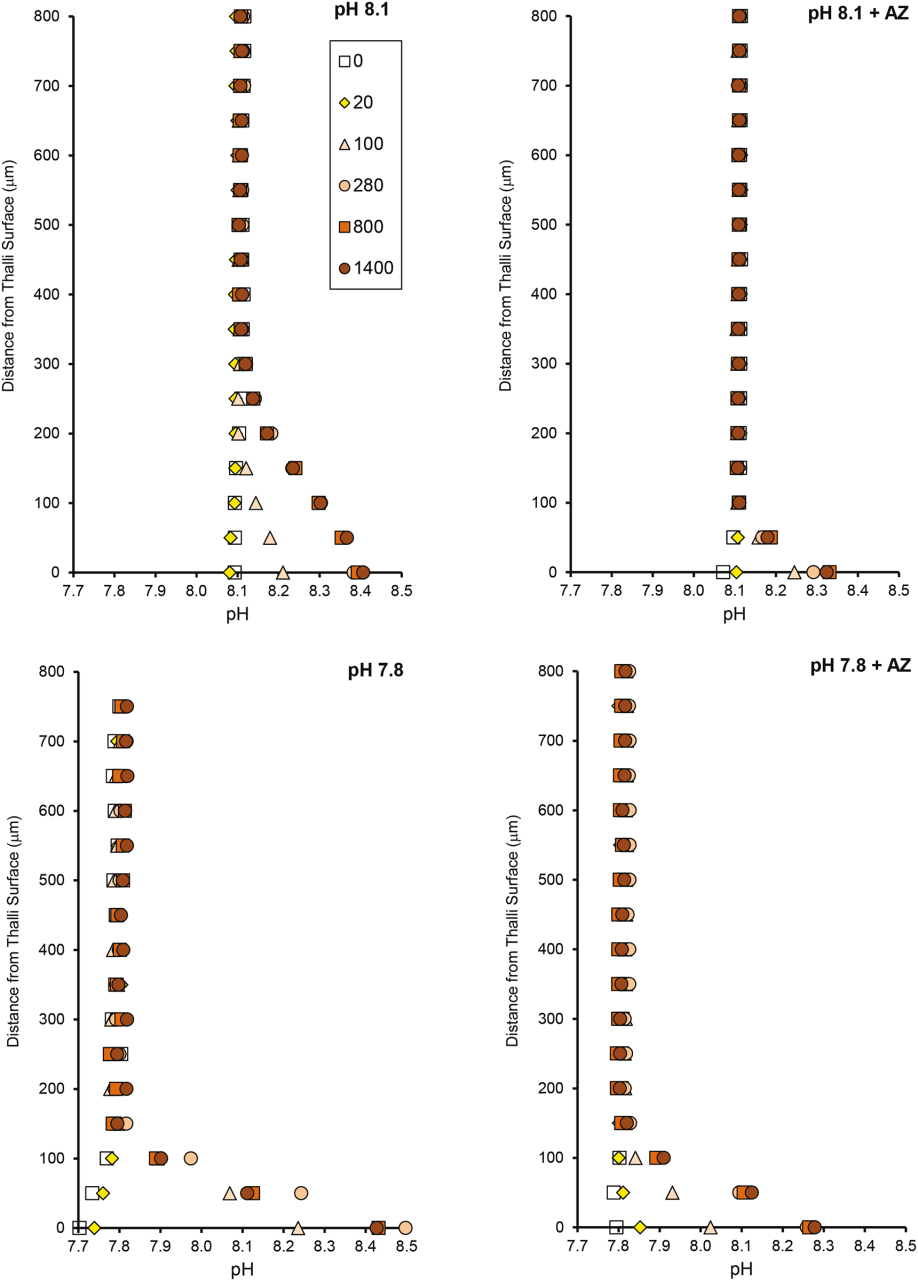
Seawater pH and AZ affect surface pH at varying light. pH profiles from a single rock (CCA I) measured 0–800 μm from the thallus surface at two pH levels (8.12, 7.80) in the absence and presence (+ AZ) of the carbonic anhydrase inhibitor acetazolamide (AZ). Profiles were measured at light intensities from 0–1400 μmol photons m^-2^ s^-1^ (0: open squares, 20: yellow diamonds, 100: orange triangles, 280: orange circles, 800: dark orange squares, 1400: red circles). A similar pattern was found for CCA II.

**Fig 7:**
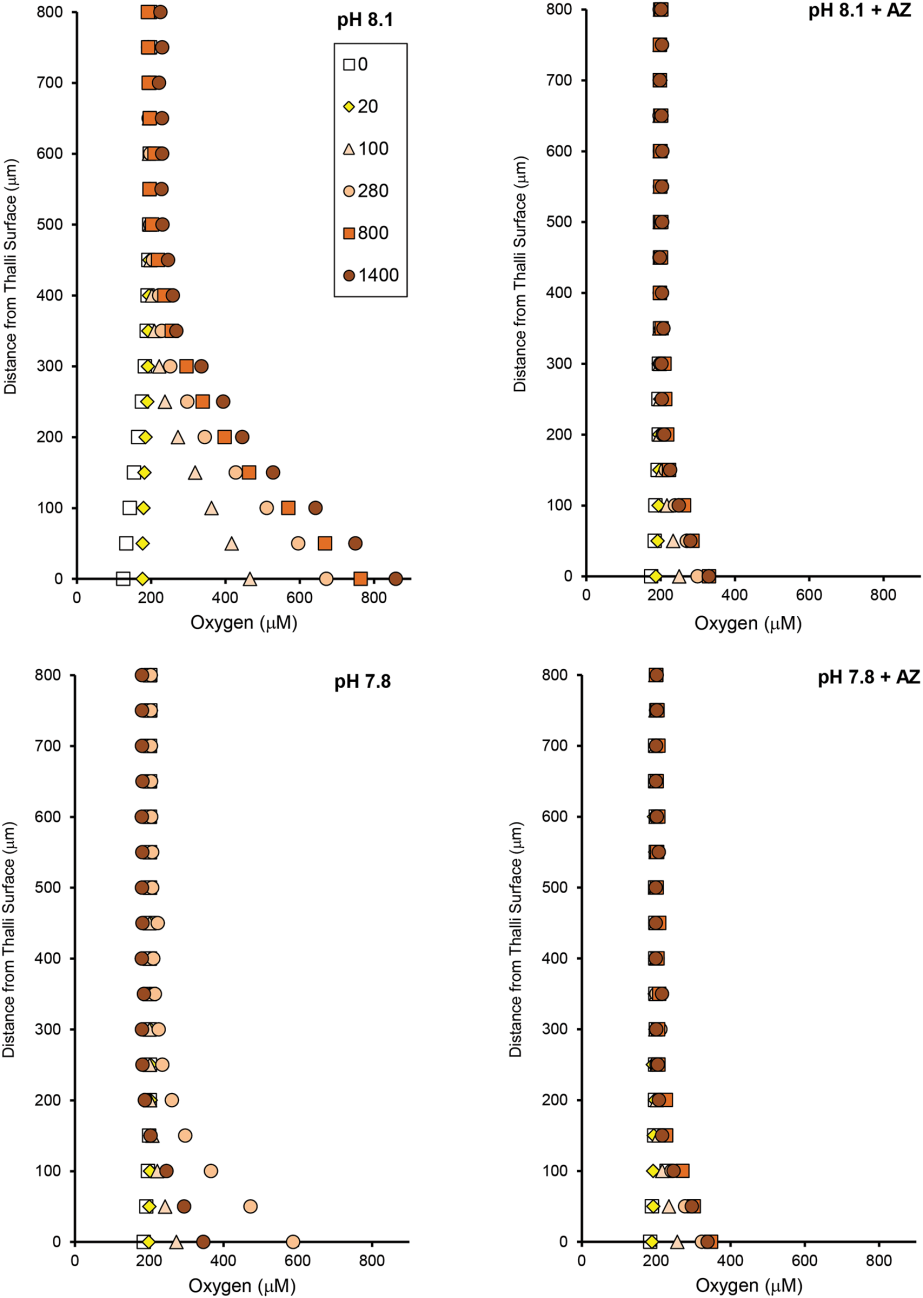
Seawater pH and AZ affect surface O_2_ at varying light. Oxygen profiles from a single rock (CCA I) measured 0–800 μm from the thallus surface at two pH levels (8.12, 7.80) in the absence and presence (+ AZ) of the carbonic anhydrase inhibitor acetazolamide (AZ). Profiles were measured at light intensities from 0–1400 μmol photons m^-2^ s^-1^ (0: open squares, 20: yellow diamonds, 100: orange triangles, 280: orange circles, 800: dark orange squares, 1400: red circles). A similar pattern was found for CCA II.

**Fig 8:**
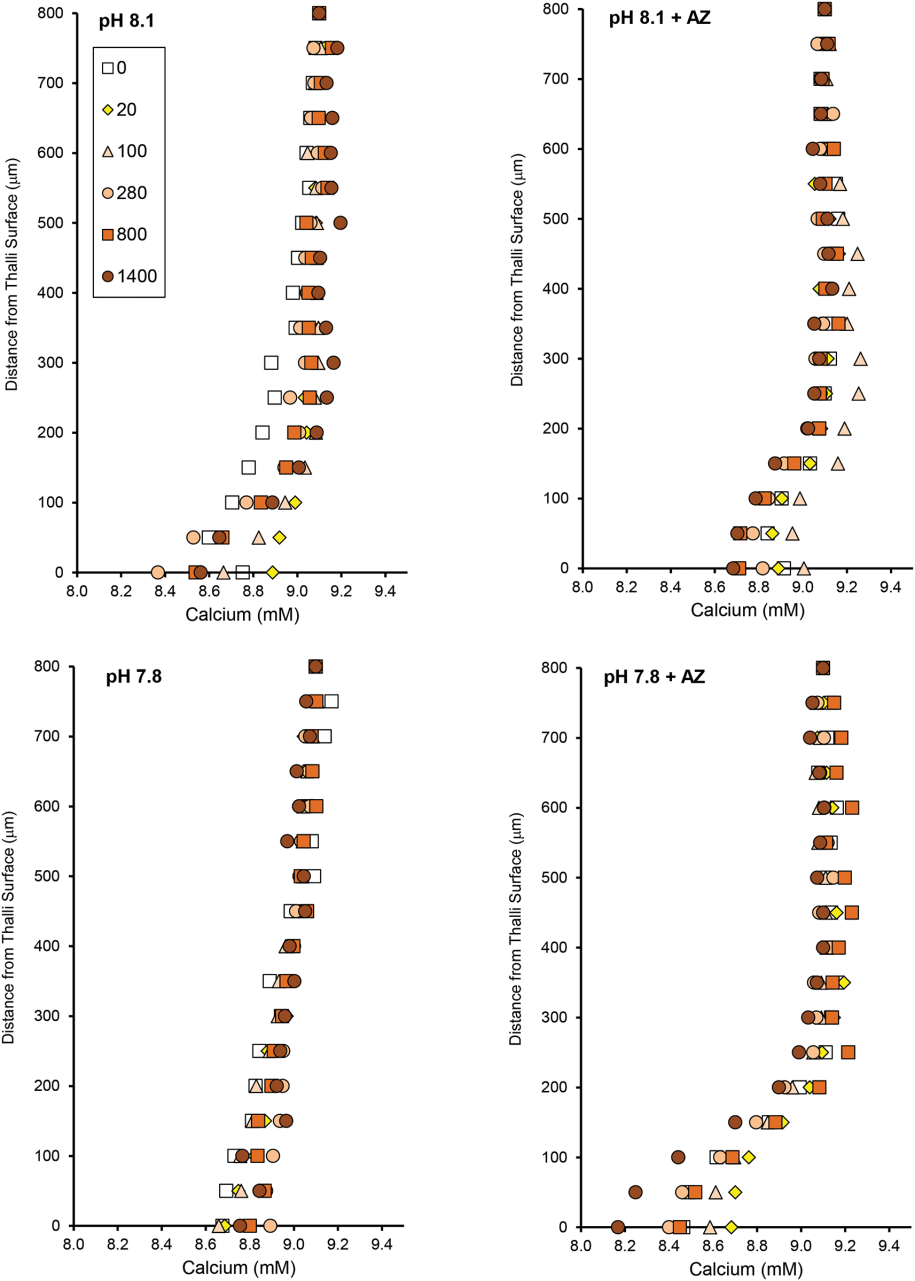
Seawater pH and AZ affect surface Ca^2+^ at varying light. Calcium profiles from a single rock (CCA I) measured 0–800 μm from the thallus surface at two pH levels (8.12, 7.80) in the absence and presence (+ AZ) of the carbonic anhydrase inhibitor acetazolamide (AZ). Profiles were measured at light intensities from 0–1400 μmol photons m^-2^ s^-1^ (0: open squares, 20: yellow diamonds, 100: orange triangles, 280: orange circles, 800: dark orange squares, 1400: red circles). A similar pattern was found for CCA II.

Profiles of pH, O_2_, and Ca^2+^ showed that the diffusive boundary layer above the surface of the algae ranged from 100–300 μm at the experimental flow rate (Figs 6–8). The variation in DBL thickness likely depended on the shape of the individual CCA, which depended on the substratum upon which it was growing. The individuals growing on a flat surface had thin boundary layers, while those growing on a more heterogeneous substratum had thicker boundary layers.

The surface pH and O_2_ concentration determined from the profiles increased with increasing light intensity, while there was no clear trend between irradiance and Ca^2+^ concentration. The surface pH was elevated relative to seawater to the same extent at both pH treatments in the light, but in the dark, the surface pH was 0.4 units lower in the pH 7.80 treatment than in the pH 8.12 treatment (Fig 6).

### Inhibition of Inorganic Carbon Uptake Pathways

The addition of AZ decreased the boundary layer thickness of O_2_ and pH, but not Ca^2+^, and decreased the ability of the CCA to elevate O_2_ and pH at its surface relative to the seawater. In contrast, Ca^2+^ concentration at the surface and the resulting fluxes did not show a clear pattern with respect to AZ, and the responses to both factors varied between individuals.

**Fig 9:**
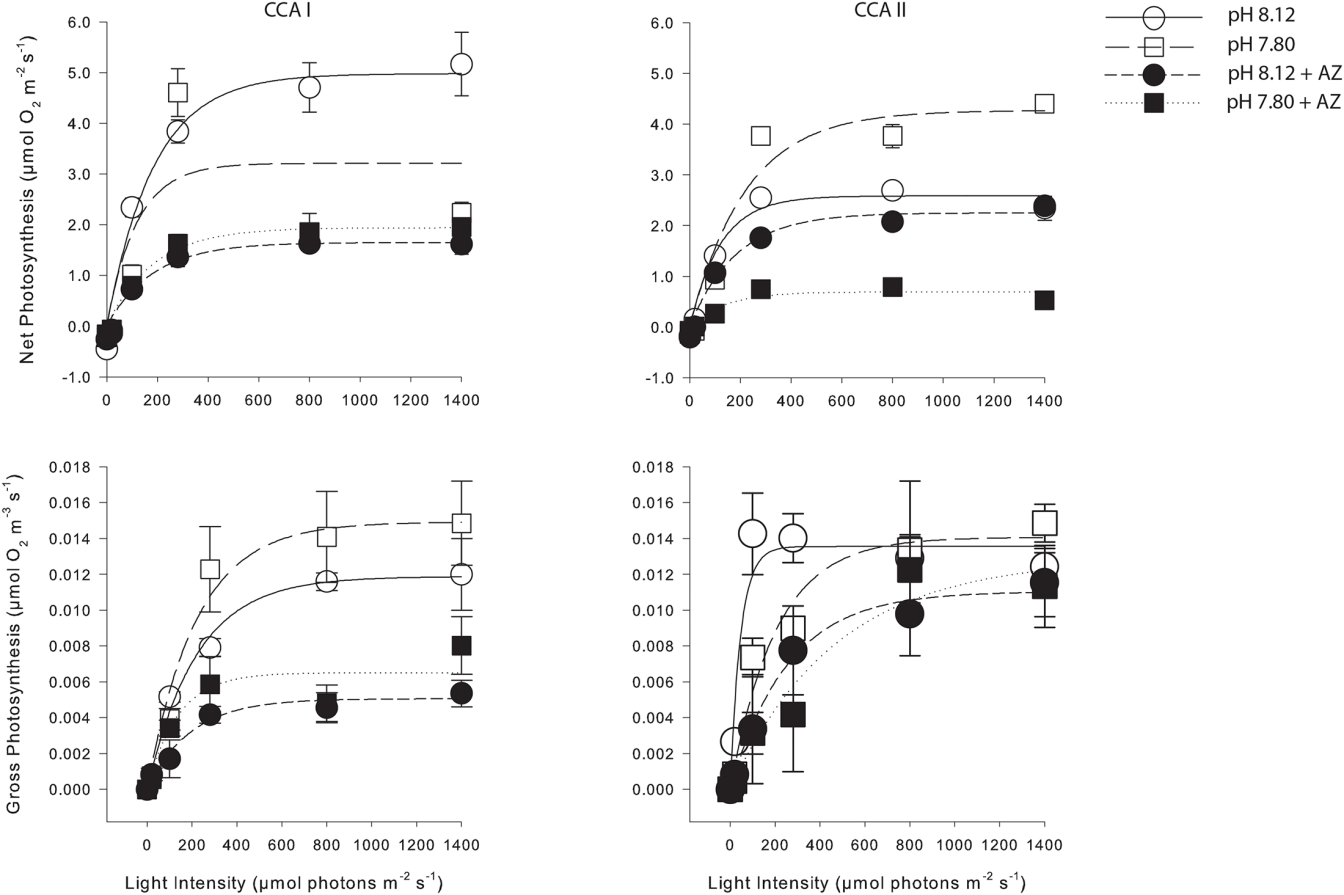
Seawater pH and AZ affect net and gross photosynthesis. Net and gross photosynthesis rates as a function of light intensity of two specimens (CCA I, CCA II) exposed to two seawater pH levels (8.12, 7.80) and with and without the carbonic anhydrase inhibitor acetazolamide (AZ). Data are the mean ± standard error of multiple measurements made on the same individuals. Open circles and smooth lines: pH 8.12, filled circles and short dashed line: pH 8.12 with AZ, open squares and long dashed line: pH 7.80, filled squares and dotted line: pH 7.8 with AZ. Non-linear regression analysis was conducted using Sigma Plot 12.0 and the equation y = a*(1-exp(-b*x)).

Inhibition of CA_ext_ by AZ decreased the slope of the O_2_ and pH (and hence H^+^) dynamics (Table 1). Acetazolamide also decreased the net and gross photosynthetic rates in both pH treatments and at all light levels investigated (Fig 9, Table 2). Net photosynthesis was inhibited by 59 ± 10% at pH 8.12 and 67 ± 13% at pH 7.8 in the light. Gross photosynthesis was inhibited 50 ± 8% and 39 ± 7% at pH 8.12 and pH 7.8, respectively. There was no clear relationship between light intensity and extent of photosynthesis inhibition by AZ due to high variability between individuals. The light compensation points (light intensity where net photosynthesis = respiration) were lowest (10 and 17 μmol photons m^-2^ s^-1^ for CCA I and CCAII, respectively) for algae in control conditions (pH 8.12, no AZ), and increased to 20–30 when exposed to low pH and/or AZ (Table 2). As with the profiles, Ca^2+^ fluxes were highly variable between the two individuals, and did not show a clear response to pH or AZ (Table 3). Nevertheless, partial inhibition of photosynthesis with AZ did not inhibit Ca^2+^ uptake at the thallus surface, suggesting that photosynthesis and Ca^2+^ uptake are not necessarily directly linked. Comparison of the Ca^2+^ fluxes with *in situ* estimates of calcification from the literature show similar results. At our highest irradiance investigated, Ca^2+^ fluxes ranged from 5–12 mmol Ca^2+^ m^-2^ hr^-1^, which is similar to the rate reported by [53] (9.5 mmol CaCO3 m^-2^ hr^-1^) for *Porolithon onkodes*.

**Table 1:** The rate of change in oxygen and proton concentration at the thalli surface occurring within the first ten seconds of turning on or off the light at photosynthetic saturating (800 μmol photons m^-2^ s^-1^) and sub-saturating (100 μmol photons m^-2^ s^-1^) light intensities, two seawater pH levels (8.12 or 7.80) and in the absence and presence of acetazolamide (AZ). Data are means ± SD of multiple measurements from CCAII.

Light	AZ	pH	O_2_	H^+^
Level	(+/-)		(nmol m^-3^ s^-1^)	(nmol m^-3^ s^-1^)
*Change after Light On*				
800	-AZ	8.12	8.2 ± 0.97	-3.7E-05 ± 2.0E-06
800	+AZ	8.12	4.6 ± 2.1	-1.1E-05 ± 5.4E-06
800	-AZ	7.8	7.4 ± 3.8	-7.3E-05 ± 2.3E-05
800	+AZ	7.8	nd	nd
100	-AZ	8.12	5.5 ± 0.04	-2.9E-05 ± 1.8E-06
100	+AZ	8.12	2.8 ± 1.4	-0.72E-05 ± 1.1E-06
100	-AZ	7.8	3.8 ± 0.55	-2.8E-05 ± 9.3E-06
100	+AZ	7.8	1.7 ± 0.62	-2.6E-05 ± 6.2E-07
*Change after Light Off*				
800	-AZ	8.12	-11.0 ± 1.1	4.1E-05 ± 4.6E-06
800	+AZ	8.12	-6.3 ± 0.37	2.6E-05 ± 2.6E-06
800	-AZ	7.8	-12.0 ± 3.1	8.7E-05 ± 7.5E-06
800	+AZ	7.8	nd	nd
100	-AZ	8.12	-7.0 ± 0.47	2.7E-05 ± 2.9E-06
100	+AZ	8.12	-4.6 ± 0.98	1.0E-05 ± 2.9E-06
100	-AZ	7.8	-4.6 ± 0.18	5.4E-05 ± 2.6E-06
100	+AZ	7.8	-1.8 ± 0.45	5.3E-05 ± 4.9E-06

nd = not determined because sensor broke.

**Table 2:** Photosynthetic parameters (maximum net photosynthetic rate, Pmax, photosynthetic efficiency, alpha, and light compensation points, Ic) based on non-linear regression analysis of the photosynthesis—irradiance curves for both CCA I and CCA II at two pH levels (8.12, 7.80) and in the absence and presence of the carbonic anhydrase inhibitor, acetazolamide.

AZ	pH	CCA	P_max_	alpha	I_c_
(+/-)		(I/II)			
-AZ	8.12	I	5	0.005	17
+AZ	8.12	I	1.7	0.006	30
-AZ	7.8	I	3.2	0.008	30
+AZ	7.8	I	1.9	0.005	20
-AZ	8.12	II	2.6	0.008	10
+AZ	8.12	II	2.3	0.006	20
-AZ	7.8	II	4.3	0.005	20
+AZ	7.8	II	0.69	0.007	23

P_max_ (μmol O_2_ m^-2^ s^-1^); alpha (μmol O_2_ μmol photons^-1^); I_c_ (μmol photons m^-2^ s^-1^)

**Table 3:** Calcium fluxes (mol Ca^2+^ m^-2^ s^-1^) at all experimental light levels (μmol photons m^-2^ s^-1^) and two pH levels (8.12, 7.80) in the absence and presence (+AZ) of acetazolamide. Fluxes were calculated from profiles in Fig 8 (CCA I; top) and additional profiles from CCA II (bottom, profiles not shown). Data are means ± SE of multiple measurements from each CCA.

Light Level	pH 8.12	pH 8.12 + AZ	pH 7.80	pH 7.80 + AZ
0	1.8E-06 ± 2.5E-07	0.80E-06 ± 19E-07	-0.11E-06 ± 7.9E-07	1.8E-06 ± 11E-07
20	1.0E-06 ± 3.3E-07	1.4E-06 ± 6.6E-07	0.16E-06 ± 3.8E-07	0.80E-06 ± 5.8E-07
100	2.8E-06 ± 5.8E-07	0.99E-06 ± 18E-07	-0.18E-06 ± 14E-07	1.1E-06 ± 9.5E-07
280	4.1E-06 ± 5.0E-07	0.12E-06 ± 10E-07	0.27E-06 ± 6.5E-07	2.4E-06 ± 11E-07
800	3.0E-06 ± 17E-07	0.35E-06 ± 4.5E-07	1.4E-06 ± 15E-07	2.4E-06 ± 4.7E-07
1400	3.3E-06 ± 12E-07	0.11E-06 ± 10E-07	1.0E-06 ± 12E-07	2.8E-06 ± 8.8E-07
0	2.2E-06 ± 4.6E-07	0.89E-06 ± 2.1E-07	3.1E-06 ± 4.1E-07	1.4E-06 ± 5.5E-07
20	2.1E-06 ± 1.6E-07	3.4E-06 ± 1.4E-07	3.8E-06 ± 6.4E-07	2.2E-06 ± 2.7E-07
100	1.9E-06 ± 4.9E-07	8.5E-06 ± 4.7E-07	5.5E-06 ± 7.3E-07	6.5E-06 ± 2.5E-07
280	1.5E-06 ± 2.7E-07	8.9E-06 ± 9.3E-07	6.3E-06 ± 12E-07	6.5E-06 ± 4.8E-07
800	0.49E-06 ± 1.5E-07	9.4E-06 ± 3.1E-07	7.8E-06 ± 19E-07	nd
1400	1.3E-06 ± 3.2E-07	7.4E-06 ± 8.1E-07	7.3E-06 ± 3.2E-07	9.0E-06 ± 12E-07

nd = not determined

**Fig 10:**
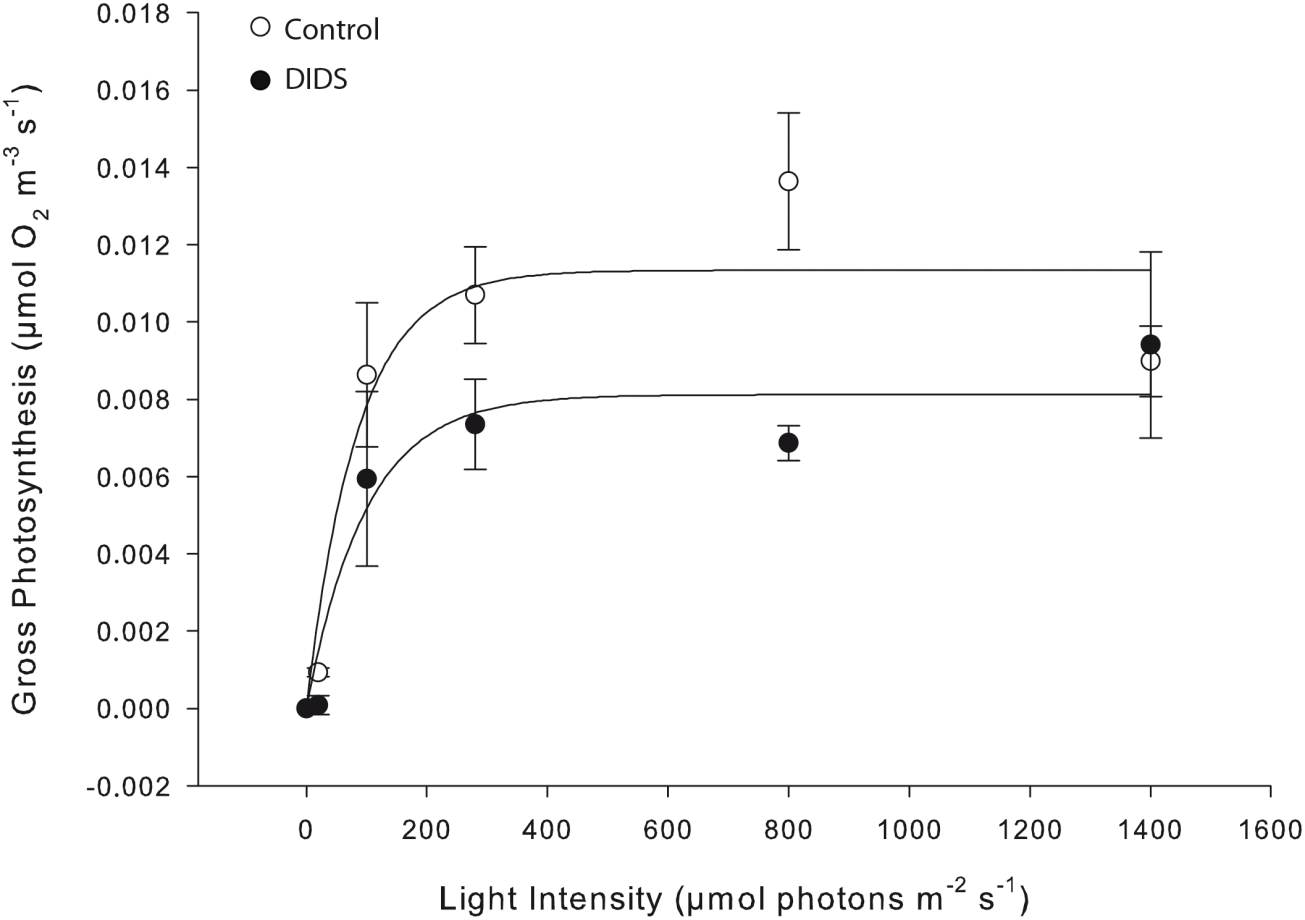
DIDS inhibits gross photosynthesis. Gross photosynthesis rates estimated using the light-dark shift method before (control) and after (DIDS) addition of the cellular anion permeability inhibitor 4,4′ diisothiocyanatostilbene-2,2′-disulphonate (DIDS). Data are the means ± SE of several measurements on a single individual (CCA III). Non-linear regression analysis was conducted using Sigma Plot 12.0 and the equation y = a*(1-exp(-b*x)).

The inhibition of anion transport by DIDS at ambient pH resulted in decreased gross photosynthesis at all light intensities investigated with the exception of the highest light intensity, 1400 μmol photons m^-2^ s^-1^, where no inhibition was detected (Fig 10). Inhibition was highest at the compensation point (90% at 20 μmol photons m^-2^ s^-1^), and ranged from 31–50% between 100–800 μmol photons m^-2^ s^-1^. When both AZ and DIDS are considered together, inhibition of gross photosynthesis at ambient pH was greater than 100% at limiting light intensities, and ranged from 77–92% at saturating light intensities, suggesting that diffusive CO_2_ entry into the algal cell contributes less than 23% of the total inorganic carbon uptake.

## Discussion

**Fig 11:**
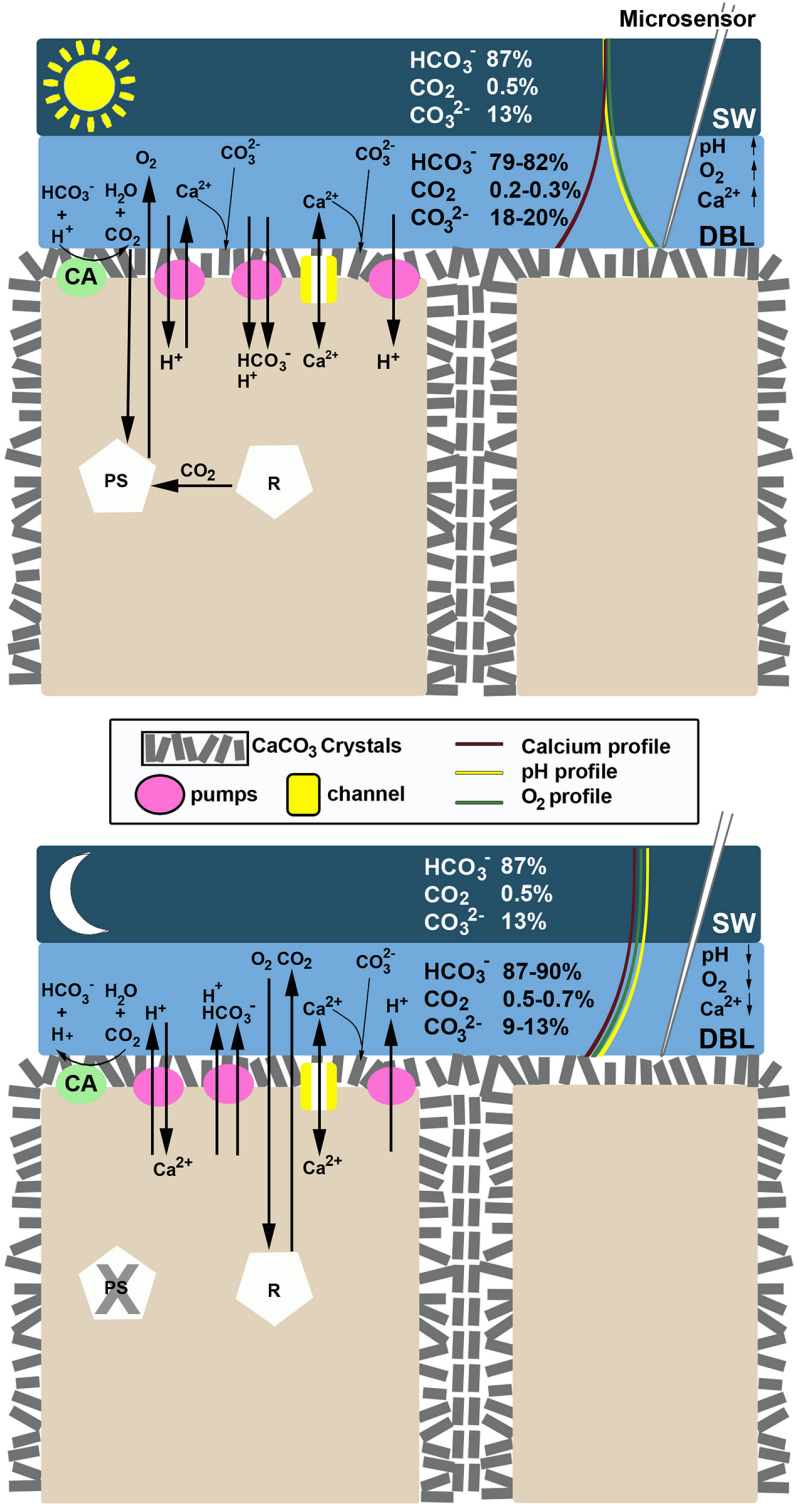
Cell model of physiological pumps involved in photosynthesis and calcification in a tropical CCA. A conceptual model showing the various pumps (pink circles), channels (yellow square) and enzymes (green circle, CA = external carbonic anhydrase) involved in photosynthesis and calcification under illuminated (top panel) and darkened (bottom panel) conditions based on the results of this study. The percentage of total dissolved inorganic carbon made up by each of the carbon species (HCO_3_^-^, CO_2_, CO_3_^2-^) are shown in the bulk seawater (SW) and diffusional boundary layer (DBL; values are approximated averages, since the DBL has a concentration gradient). The red, green and yellow curved lines penetrating through the DBL show the general trends of Ca^2+^, O_2_ and pH, respectively, under illuminated and darkened conditions as determined from our profile measurements. The arrows next to pH, O_2_ and Ca^2+^ indicate the pattern that was observed in the respective concentrations during dynamic measurements.

The results of this study indicate that this tropical CCA has the potential to metabolically control the pH, O_2_ and Ca^2+^ concentration at the surface of its thalli. We have constructed a model (Fig 11) of the hypothesized mechanisms based on our observations (Table 4). The dynamics of pH and oxygen corresponded to photosynthetic activity, as both parameters increased in the light, and decreased in the dark during respiration. However, the speed of the pH dynamics and the lack of complete inhibition of pH dynamics in the presence of DCMU provides evidence that pH changes at the thallus surface were not simply a result of photosynthesis or respiration-induced changes in carbonate chemistry, but also a light-induced proton pump that is independent from photosynthesis. De Beer and Larkum [54] also found that pH dynamics at the thallus surface of the calcifying Chlorophyte, *Halimeda discoidea*, were controlled by a separate light-dependent processes in addition to photosynthesis. The results of our study demonstrate that this tropical CCA has the ability to actively control the influx and efflux of protons and, thus, thallus surface pH. This biological control may potentially facilitate calcification, even under low pH. Furthermore, protons pumped into the cell in the light may provide a proton source for the internal CA enzyme that provides CO_2_ to the carbon fixing enzyme rubsico.

**Table 4:** Proposed biotic controls of CCA surface pH and Ca^2+^-H^+^ exchange processes based on dynamic studies (D) and profiles (P) at the thallus surface of a tropical CCA.

Biotic Process	Evidence	Method
*pH and Oxygen*		
Photosynthesis partially controlled surface pH	pH and O_2_ dynamics corresponded	D
	ΔpH and O_2_ fluxes corresponded with and without inhibitors	P
Photosynthesis principally dependent on HCO_3_^-^	Inhibitors of HCO_3_^-^ uptake lowered photosynthesis >77%	P
	pH change lower than theoretical solely based on CO_2_ use	P
Cellular H^+^ pumping	Steady state reached rapidly (< 1min) in light/dark transitions	D
Non-photosynthetic light-dependent H^+^ pump	H^+^ uptake in the light despite inhibited photosynthesis	P
pH control at thalli surface by proton pumping	ΔpH at low pH_SW_ greater than ambient in the light	P
	surface pH similar in low/ambient pH seawater in the light	P
	H^+^ efflux greater at low pH in dark	P
*pH and Calcium*		
Ca^2+^-ATPase presence	Ca^2+^ and pH dynamics most often increased in the light,	
	decreased in the dark	D
Ca^2+^ channel mediated flux	Uncoupling of Ca^2+^ and H^+^ fluxes in presence of AZ	P
	Opposite trend of Ca^2+^ and pH dynamics observed	D
Non-photosynthetic net Ca^2+^ uptake	Ca^2+^ flux did not correspond to irradiance	P

Through the use of the non-permeable carbonic anhydrase inhibitor AZ and the anion exchange inhibitor DIDS, we were able to characterize some major features of the carbon concentrating mechanism of a tropical CCA. Our results suggest that diffusive CO_2_ uptake contributes less than 23% of total inorganic carbon uptake, and that both CA-mediated hydrolysis of HCO_3_^-^ to CO_2_ and direct uptake of HCO_3_^-^ into the cell contribute greater than 77% of inorganic carbon uptake in this alga. This result is consistent with the fact that the observed delta pH was lower than what would be expected due to sole CO_2_ use at ambient seawater pH conditions, following calculations from [55]. Nevertheless, the effects of DIDS on photosynthesis should be interpreted cautiously, as DIDS may affect other anion transport processes that are not directly related to HCO_3_^-^ transport. Although other coralline algae have been shown to use HCO_3_^-^ as an inorganic carbon source [56–57], our data do not support the notion for this species that HCO_3_^-^ uptake is facilitated by the secretion of H^+^ outside of the plasma membrane, creating regions of low pH/high CO_2_ [57], as we always measured elevated surface pH in the light.

Exposure of this CCA to low seawater pH (7.80) demonstrated its potential to regulate the pH and hence proton concentration at the surface of its thallus, so that the aragonite saturation state always remained above one, even at pH 7.8 in the dark (see S1 Table). The surface pH in the light reached the same elevated pH (ca. 8.4) regardless of experimental seawater pH. Thus, additional proton pumping into the cell was required at low pH in order to reach the same surface pH, suggesting strong biotic control of ion transport. Furthermore, in the dark, the delta pH was greater at seawater pH 7.80 than at pH 8.12 in the absence of AZ, suggesting that more protons were pumped out of the cell in the lower pH treatment. It is not clear what caused the small increase in surface pH in the dark at pH 7.80 in the presence of AZ. A lower surface pH in the dark when exposed to low seawater pH may have negative repercussions for calcification and lead to potentially higher dissolution rates of tropical CCA under future ocean condition. These results complement recent literature predicting that high proton concentrations, and hence changing proton gradients, rather than carbonate ion limitation, will control the response of calcifying organisms to ocean acidification [58–59].

The calcium fluxes observed at the surface of the CCA thallus were large, ranging from 0.5 to 10 μmol m^-2^ s^-1^, with the upper values exceeding the O_2_ fluxes under saturating light, which ranged from 2–5 μmol O_2_ m^-2^ s^-1^. This high ratio of Ca^2+^ to O_2_ fluxes is consistent with those reported in coccolithophores, where molar fluxes of calcium and inorganic carbon into the coccolith vesicle can equal the molar flux of photosynthetically fixed carbon [60]. In addition to being large, the Ca^2+^ fluxes often mirrored the pH gradient (and hence changes in protons). In the most frequently observed dynamics, Ca^2+^ efflux in the light and proton influx into the cell were observed (i.e. surface pH and Ca^2+^ increased). In the dark, Ca^2+^ concentration decreased at the surface, corresponding to an influx into the cell, while protons were pumped out of the cell (surface pH decreased). The calcium transported into the cell may have been stored in the vacuole, which plays a pivotal role in H^+^ and Ca^2+^ storage inside the cells of photosynthetic eukaryotes [61], and some Ca^2+^ may have been directly incorporated into cell wall or interperithallium crystals, as suggested by [12] in *Clathromorphum*. We suggest that there may be a Ca^2+^-ATPase present, which exchanges Ca^2+^ for H^+^. Ca^2+^-ATPase has been reported among several calcifying organisms, including corals and algae [62] and, specifically relevant for the Corallinaceae [63]. Furthermore, cytoplasmic Ca^2+^-H^+^ exchange has been reported in non-calcifying algae [64], and several species of Rhodophytes whose genomes have been sequenced show evidence of genes for Ca^2+^-ATPase and Ca^2+^-H^+^ exchangers [61].

We did not observe a strong light effect in the Ca^2+^ profiles or calculated Ca^2+^ fluxes, in contrast to the dark/light dynamics. This may be explained by the differences in spatial and temporal scales between the two measurements. The dynamics measured at the thalli surface captured the light/dark gross cellular processes occurring over time, while profiles captured snapshots of diffusional fluxes at a single point in time. Nevertheless, the dynamics showed that surface Ca^2+^ concentration was always lower than in the seawater, and the profiles always showed Ca^2+^ uptake relative to the seawater. Furthermore, we did not see a correlation between Ca^2+^ fluxes and photosynthesis, suggesting that calcification in this species, or at least Ca^2+^ uptake, is not simply driven by photosynthesis. There is strong evidence based on SEM that calcification and decalcification can be occurring simultaneously in CCA, as decalcification is required for new cellular growth and recalcification [25,65], although these processes likely occur on a longer time scale than what we could measure with microsenesors. Our results from Ca^2+^ microsensor measurements provide an important contribution to the literature on CCA calcification, because we show that even at pH_SW_ 7.8, net dissolution at the growing surface does not occur. Previous studies have not been able to separate the contributions from changes in photosynthesis versus dissolution of exposed skeleton (e.g. [41]) (i.e. along broken edges) as we have done in our study using microsensors.

When CA was inhibited with AZ, calcium profiles showed an increase in Ca^2+^ uptake (a decrease in surface Ca^2+^ concentration for both individuals at pH 7.8, and for CCA II at pH 8.1), but the pH gradient decreased. The surface pH and Ca^2+^ fluxes were closely coupled without CA_ext_ inhibition, but there seemed to be a decoupling in the presence of AZ. Therefore, it cannot be ignored that the Ca^2+^ fluxes likely involve calcium channels, in addition to Ca^2+^-ATPase. The blockage of electron transport by inhibitors has been shown to cause a transient hyperpolarization of the plasma membrane in plants, which drives the uptake of divalent cations [66]. This uptake of cations also occurs in the dark, when chloroplasts release Ca^2+^ into the cell, resulting in a transient hyperpolarization of the plasma membrane, which then drives a release of salts coupled with an uptake of divalent cations; hence, a Ca^2+^-release-induced Ca^2+^ uptake triggered by darkening [66]. This mechanism would explain why we saw higher calcium uptake at the algal surface in the measured profiles in the presence of AZ, despite a decrease in the pH gradient. It is possible that Ca^2+^ fluxes related to signaling and Ca^2+^ uptake for calcification are separated in this alga, as in coccolithophores [60,67]; however, much more work is required to validate these complex mechanisms of Ca^2+^ flux.

Further evidence for the complexity of Ca^2+^ dynamics in this alga is shown by our observation on several occasions of a simultaneous efflux of Ca^2+^ and H^+^ in the dark and influx in the light. We hypothesize that this observation may be due to temporal variability linked to the growth mechanism of this alga, which involves localized decalcification and eventual sloughing-off of the epidermal cells [68,69]. In fact, decalcification of the cell walls during epithallial cell turnover has been reported in CCA [27], and could explain the temporal variability in Ca^2+^ dynamics that we measured, as decalcification would require an abundance of protons and produce an excess of Ca^2+^ ions outside the cell.

Overall, our data support the contention that calcification is not simply dependent on CO_2_-removal by photosynthesis in CCA, but also metabolically controlled through photosynthesis-independent ion pumps and channels [4,12], as demostrated in this tropical coralline alga (S1 Table). Such a mechanism is partially complementary to the calcification scheme suggested by [42] for *P*. *onkodes* (formerly *Hydrolithon onkodes*), as we saw an increase in [OH^-^] with increasing photosynthetic rates, as well as H^+^ release in the dark and uptake in the light, based on surface pH data. We were unable to confirm the presence of a HCO_3_^-^/H^+^ symporter from our data, but it is likely that an anion transporter is present, as shown by the inhibition of photosynthesis in the presence of DIDS. We further showed that proton pumps and perhaps H^+^/Ca^2+^ exchangers are controlling H^+^ fluxes into and out of the cells.

In conclusion, tropical CCA have the capacity to strongly influence the thallus surface chemistry through multiple processes, including active H^+^, CO_2_ and HCO_3_^-^ uptake during photosynthesis, CO_2_ and H^+^ release during respiration, a photosynthesis-independent proton pump, and light-triggered H^+^ and Ca^2+^ exchange (Table 4). There are likely several processes controlling Ca^2+^ fluxes, which are complex and show temporal variability. The combination of an active carbon concentrating mechanism, a photosynthesis-independent, light-induced H^+^ pump, and carefully regulated Ca^2+^ fluxes suggest that this CCA has the potential to dictate strict control over its surface chemistry, and consequently the chemical environment where calcification occurs. However, it is not clear if this alga will be able to compensate for the low surface pH under dark conditions and the additional energy requirements potentially needed for increased H^+^ pumping under low seawater pH conditions that are predicted to occur in future surface oceans. The complexity of ion transport systems involved in photosynthesis and calcification observed in this study reveal that it is necessary to gain a deeper understanding of these processes in CCA in order to be able to predict the potential response of these important autotrophs to global change.

## Supporting Information

S1 Fig Scanning electron microscopy images. Additional scanning electron images of the epithelium surface (A,B) and cross sections (C,D) of the CCA used in our microsensor studies that are not depicted in Fig 1a. A) 1611x, B) 6442x, C) 3221x, D) 7813x.
(DOCX)

S1 Table Surface seawater chemistry calculations. Surface seawater chemistry calculated for CCA I and II using measured O_2_ concentration differences between the seawater and the thallus surface (ΔO_2_) and measured surface pH (measured values indicated in bold). The ΔHCO_3_^-^ was calculated using Equation 4. Surface DIC (μM) was calculated by subtracting the DIC consumed at the surface (ΔHCO_3_^-^) from the DIC concentration measured in the bulk seawater for each pH treatment (pH 8.1 = 2165, pH7.8 = 2379). The remaining carbonate chemistry parameters, listed on the right side of the table, were calculated in CO2Sys v2.1 using the NBS pH scale and K1 and K2 from Mehrbach et al. (1973) refit by Dickson and Millero (1987). TA, HCO_3_^-^, CO_3_^2-^, OH^-^ (μmol kg SW^-1^), pCO_2_ (μatm), Ω_Ar_ = saturation state of aragonite. Dashes indicate missing data. The lowest Ω_Ar_ values are shown in italics.
(DOCX)
