# Supplementary material for: Biotic Control of Surface pH and Evidence of Light-Induced H+ Pumping and Ca2+-H+ Exchange in a Tropical Crustose Coralline Alga
Source: PLoS One. 2016 Jul 26;11(7):e0159057. doi: 10.1371/journal.pone.0159057 (PMC4961294; doi:10.1371/journal.pone.0159057)

S2 Fig. Additional scanning electron images of the epithelium surface (A,B) and cross sections (C,D) of the CCA used in our microsensor studies that are not depicted in Fig. 1a. A) 1611x, B) 6442x, C) 3221x, D) 7813x.


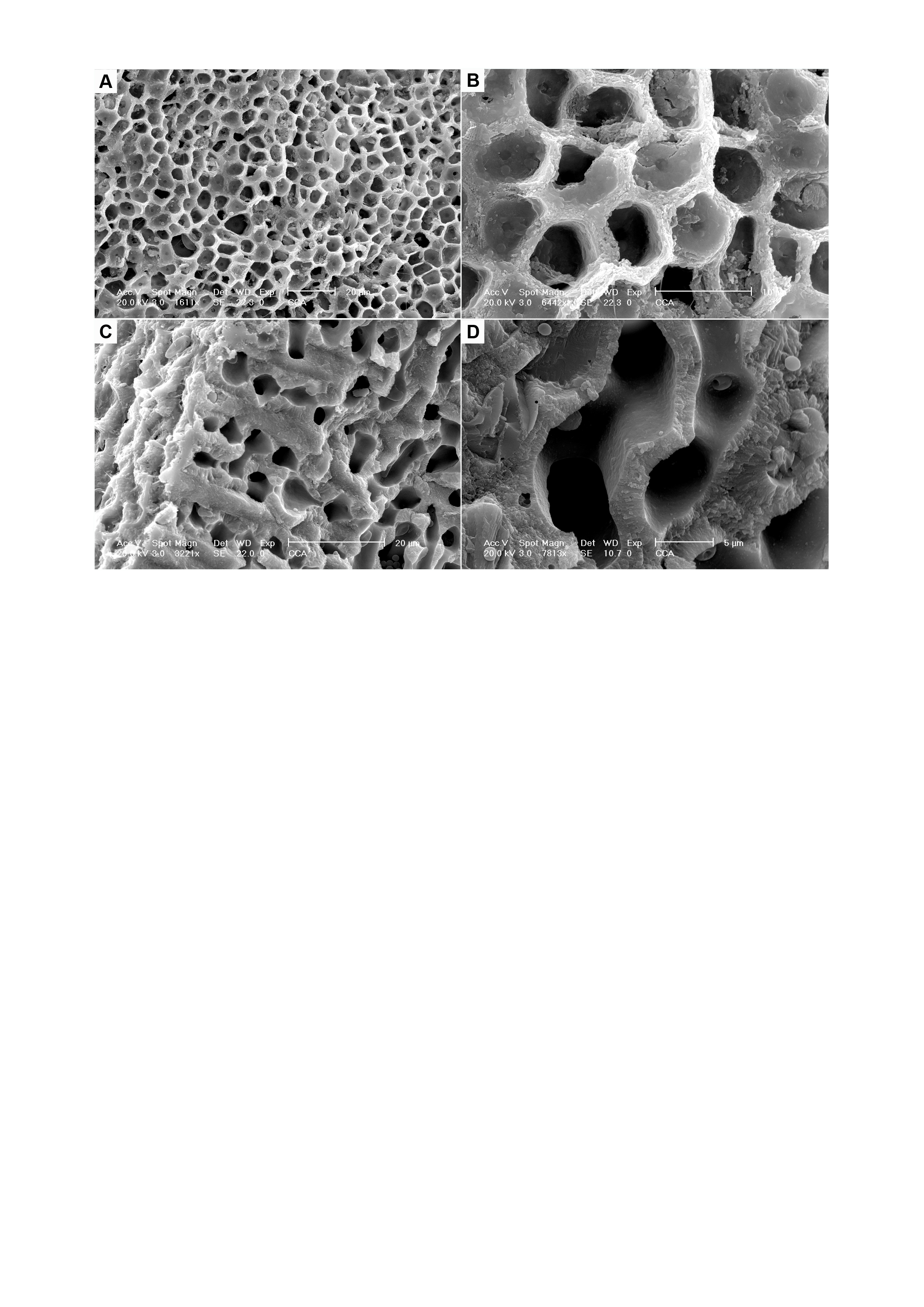

Supplement: S1 Fig — Additional scanning electron images of the epithelium surface (A,B) and cross sections (C,D) of the CCA used in our microsensor studies that are not depicted in Fig 1a. A) 1611x, B) 6442x, C) 3221x, D) 7813x. (DOCX) [file pone.0159057.s001.docx]
