# Supplementary material for: Biotic Control of Surface pH and Evidence of Light-Induced H+ Pumping and Ca2+-H+ Exchange in a Tropical Crustose Coralline Alga
Source: PLoS One. 2016 Jul 26;11(7):e0159057. doi: 10.1371/journal.pone.0159057 (PMC4961294; doi:10.1371/journal.pone.0159057)
Supplement: S1 Table — Surface seawater chemistry calculated for CCA I and II using measured O2 concentration differences between the seawater and the thallus surface (ΔO2) and measured surface pH (measured values indicated in bold). The ΔHCO3- was calculated using Equation 4. Surface DIC (μM) was calculated by subtracting the DIC consumed at the surface (ΔHCO3-) from the DIC concentration measured in the bulk seawater for each pH treatment (pH 8.1 = 2165, pH7.8 = 2379). The remaining carbonate chemistry parameters, listed on the right side of the table, were calculated in CO2Sys v2.1 using the NBS pH scale and K1 and K2 from Mehrbach et al. (1973) refit by Dickson and Millero (1987). TA, HCO3-, CO32-, OH- (μmol kg SW-1), pCO2 (μatm), ΩAr = saturation state of aragonite. Dashes indicate missing data. The lowest ΩAr values are shown in italics. (DOCX) [file pone.0159057.s002.docx]

S1. Surface seawater chemistry calculated for CCA I and II using measured O_2_ concentration differences between the seawater and the thallus surface (ΔO_2_) and measured surface pH (measured values indicated in bold). The ΔHCO_3_^-^ was calculated using Equation 4. Surface dissolved inorganic carbon (DIC, µM) was calculated by subtracting the DIC consumed at the surface (ΔHCO_3_^-^) from the DIC concentration measured in the bulk seawater for each pH treatment (pH 8.1 = 2165, pH7.8 = 2379). The remaining carbonate chemistry parameters, listed on the right side of the table, were calculated in CO2Sys v2.1 using the NBS pH scale and K1 and K2 from Mehrbach et al. (1973) refit by Dickson and Millero (1987). TA, HCO_3_^-^, CO_3_^2-^, OH^-^ (µmol kg SW^-1^), pCO_2_ (µatm), Ω_Ar_ = saturation state of aragonite. Dashes indicate missing data. The lowest Ω_Ar_ values are shown in italics.

| Rock | pH | Light | **ΔO_2_**  **(µM)** | **Surface pH** | ΔHCO_3_^-^ (µM) | Surface  DIC | TA | pCO_2_ | HCO_3_^-^ | CO_3_^2-^ | CO_2_ | OH^-^ | Ω_Ar_ |
| --- | --- | --- | --- | --- | --- | --- | --- | --- | --- | --- | --- | --- | --- |
| I | 8.1 | 0 | **-72.30** | **8.09** | -64.35 | 2229.35 | 2509.0 | 563.4 | 2005.1 | 208.7 | 15.2 | 6.4 | 3.34 |
|  |  | 20 | **-20.98** | **8.08** | -18.67 | 2183.67 | 2452.6 | 568.8 | 1969.3 | 199.4 | 15.3 | 6.2 | 3.19 |
|  |  | 100 | **271.85** | **8.21** | 241.94 | 1923.06 | 2250.7 | 360.5 | 1683.4 | 229.9 | 9.7 | 8.4 | 3.68 |
|  |  | 280 | **479.22** | **8.38** | 426.48 | 1738.52 | 2170.3 | 207.6 | 1440.9 | 292.5 | 5.6 | 12.5 | 4.69 |
|  |  | 800 | **572.27** | **8.38** | 509.30 | 1655.70 | 2073.6 | 197.7 | 1372.2 | 278.5 | 5.3 | 12.5 | 4.46 |
|  |  | 1400 | **633.19** | **8.41** | 563.52 | 1601.48 | 2027.4 | 179.7 | 1314.8 | 281.4 | 4.8 | 13.2 | 4.51 |
| I | 7.8 | 0 | **-17.45** | **7.705** | -15.53 | 2394.53 | 2489.0 | 1549.7 | 2257.1 | 96.2 | 41.7 | 2.6 | *1.54* |
|  |  | 20 | **-5.68** | **7.739** | -5.06 | 2384.06 | 2491.5 | 1423.6 | 2242.3 | 103.3 | 38.3 | 2.8 | *1.65* |
|  |  | 100 | **71.26** | **8.236** | 63.42 | 2315.58 | 2708.8 | 407.1 | 2013.7 | 291.4 | 11.0 | 8.9 | 4.67 |
|  |  | 280 | **383.27** | **8.497** | 341.10 | 2037.90 | 2630.6 | 178.3 | 1608.7 | 424.5 | 4.8 | 16.3 | 6.80 |
|  |  | 800 | **-** | **8.432** | - | - | - | - | - | - | - | - | - |
|  |  | 1400 | **165.17** | **8.427** | 147.00 | 2232.00 | 2788.9 | 236.7 | 1817.4 | 408.2 | 6.4 | 13.9 | 6.54 |
| II | 8.1 | 0 | **-12.51** | **8.25** | -11.14 | 2176.14 | 2562.8 | 368.9 | 1884.5 | 281.6 | 9.9 | 9.2 | 4.51 |
|  |  | 20 | **8.90** | **8.30** | 7.92 | 2157.08 | 2581.6 | 321.8 | 1840.5 | 307.9 | 8.7 | 10.3 | 4.93 |
|  |  | 100 | **90.20** | **8.42** | 80.27 | 2084.73 | 2609.5 | 224.7 | 1701.9 | 377.0 | 6.1 | 13.7 | 6.04 |
|  |  | 280 | **167.33** | **8.46** | 148.92 | 2016.08 | 2569.3 | 193.7 | 1616.4 | 394.4 | 5.2 | 15.1 | 6.32 |
|  |  | 800 | **178.07** | **8.45** | 158.48 | 2006.52 | 2545.7 | 199.9 | 1618.3 | 383.3 | 5.4 | 14.6 | 6.14 |
|  |  | 1400 | **160.25** | **8.48** | 142.61 | 2022.39 | 2589.6 | 187.5 | 1611.7 | 405.2 | 5.0 | 15.5 | 6.49 |
| II | 7.8 | 0 | **-10.34** | **7.79** | -9.20 | 2388.20 | 2518.9 | 1251.4 | 2237.3 | 117.0 | 33.7 | 3.2 | *1.87* |
|  |  | 20 | **-5.64** | **7.86** | -5.02 | 2384.02 | 2545.5 | 1064.2 | 2219.9 | 135.4 | 28.7 | 3.8 | 2.17 |
|  |  | 100 | **58.08** | **8.00** | 51.69 | 2327.31 | 2556.4 | 746.3 | 2129.2 | 177.7 | 20.1 | 5.2 | 2.85 |
|  |  | 280 | **173.66** | **8.06** | 154.55 | 2224.45 | 2482.4 | 611.9 | 2013.7 | 193.8 | 16.5 | 5.9 | 3.11 |
|  |  | 800 | 255.33 | 8.12 | 227.23 | 2151.77 | 2440.3 | 512.1 | 1926.1 | 211.9 | 13.8 | 6.8 | 3.39 |
|  |  | 1400 | 287.23 | 8.05 | 255.62 | 2123.38 | 2368.1 | 597.3 | 1925.4 | 181.5 | 16.1 | 5.8 | 2.91 |

Equation 4: $\Delta O2 \frac{D_{HCO3-}}{D_{O2}} = \Delta HCO3-$

Where D_HCO3-_ and D_O2_ are the diffusion coefficients of O_2_ and HCO_3_^-^ (2.35E-09 m-2 s-1, 2.09E-09), respectively. The latter was calculated according to Li and Gregory (1974) using H^+^ as the counter ion.
